# Complications associated with perioperative use of tyrosine kinase inhibitor in cytoreductive nephrectomy

**DOI:** 10.1038/s41598-019-51548-4

**Published:** 2019-10-24

**Authors:** Filipe L. F. Carvalho, Chaoyi Zheng, Kenneth Witmer, John O’neill, John H. Lynch, Keith J. Kowalczyk

**Affiliations:** 1 Department of Urology, Medstar Georgetown University Hospital, Washington, DC USA; 2 Department of Biostatistics, Bioinformatics, and Biomathematics, Lombardi Comprehensive Cancer Center, Georgetown University, Washington, DC USA

**Keywords:** Cancer, Urology

## Abstract

Recent clinical trials have investigated the benefit of combining tyrosine kinase inhibitors (TKIs) and cytoreductive nephrectomy (CN) in patients with metastatic renal cell carcinoma. Our goal is to determine whether the perioperative use of TKIs increases the postoperative morbidity following CN in renal cell carcinoma patients. We identified 627 patients with Stage IV renal cell carcinoma who underwent CN from 2007–2010 utilizing the SEER-Medicare database. Eighty-two patients treated with TKIs were matched (3:1) to 246 controls. We calculated 30- and 90-day incidence rates of postoperative complications and mortality. On unadjusted analysis, TKI use prior to CN was associated with higher overall complication rate within 30 days (HR = 2.73, 95% CI: 1.09–6.8) after surgery. On multivariate analysis, perioperative TKI use was independently associated with higher risk for postoperative complications within 30 days (HR = 2.93, 95% CI: 1.17–7.36), as well as 90 days (HR = 1.84, 95% CI: 1.02–3.32) after nephrectomy. A higher Charlson comorbidity index also emerged to represent an independent risk factor for postoperative complications within 30 days (HR = 2.41, 95% CI: 1.44–4.02) and 90 days (HR = 2.23, 95% CI: 1.51–3.29) after nephrectomy. TKI treatment was not associated with an increased postoperative mortality at 30 and 90 days after surgery. Thus, TKI treatment was associated with an increased complication rate but not overall mortality following CN. Our results suggest that renal surgeons should be aware of possibly increased complications following CN in renal cell carcinoma patients, when TKI treatment is administered.

## Introduction

The contemporary treatment of metastatic renal cell carcinoma (RCC) incorporates both surgical and systemic therapies. The standard of care for resectable primary RCC with multiple metastases and good performance status is cytoreductive nephrectomy (CN) followed by systemic therapy^1,2^. Level one evidence has shown that CN followed by interferon (INF) improves time to progression and overall survival compared to interferon alone^3,4^. In recent times, however, targeted therapies such as tyrosine kinase inhibitors (TKI) have replaced interferon for the treatment of metastatic RCC (mRCC). Results from a recent study stemming from the Surveillance, Epidemiology, and End Results (SEER) registry showed a significant increase in the use of TKI’s over the last decade^5^, being consistent with a globally increased use of these agents in mRCC^6^. Two randomized clinical trials – SURTIME and CARMENA – were launched to determine the role and timing of CN in patients with mRCC treated with TKIs. The SURTIME trial was designed to investigate whether treatment with TKIs prior to surgery improves outcomes by identifying patients whose tumors are resistant to targeted therapies and would not benefit from CN. Patients were randomized to immediate CN followed by sunitinib vs upfront treatment with sunitinib followed by CN and sunitinib (deferred nephrectomy) in patient with intermediate risk clear cell mRCC. SURTIME showed deferred CN did not significantly improved median progression free survival (PFS) and overall survival (OS)^7^. CARMENA investigated CN followed by sunitinib vs sunitinib alone, and showed noninferiority of targeted therapy alone in patients with intermediate and poor risk mRCC^8^. Both trials reported surgical complications in patients that underwent CN. In CARMINA only 15.9% of patients in CN-sunitinib group experienced Clavien-Dindo grade III or higher complications and in SURTIME the complication rates were similar in the immediate and deferred CN groups^7,8^. However, single institution series have discrepant results regarding postoperative complications associated with TKIs following nephrectomy, such as a delay in wound healing associated with VEGF-targeted therapy or intraoperative adhesions^5,9–13^. One of the largest studies on TKI treatment prior to CN showed that 66% of patients experienced perioperative complications and were significantly more likely to have multiple complications after CN^10^. Therefore, we used a large population-based database to determine whether perioperative TKI treatment increases the postoperative complications and mortality after CN in patients with metastatic RCC.

## Materials and Methods

### Data source and study population

We obtained data from 2007–2010 from the Surveillance, Epidemiology, and End Results (SEER)-Medicare database that reflects a linkage between the SEER cancer registry and Medicare claims data. The SEER registry is comprised of 17 geographical regions that each collects information on incident cancer cases (e.g. tumor site, histology and stage, date of diagnosis, vital status, demographics) within its coverage. Patient data was de-identified and written consent requirement was waived. Institutional Review Board approval was obtained.

Inclusion criteria were: 1) Kidney cancer diagnosis in patients >65 years old other than through autopsy or death certificate 2) continuous enrollment in Medicare A and B without simultaneous Health Maintenance Organization (HMO) enrollment, and 3) patients that underwent inpatient partial or total nephrectomy (ICD-9 procedure code: 55.4, 55.51, HCPCS: 50220, 50225, 50230, 50240, 50543, 50545, 50546) (N = 19,546). We further restricted the study sample to patients who had Stage IV kidney cancer (N = 627). Records of perioperative TKI use were identified from the Part D Prescription Drug Events file via National Drug Codes. An episode of TKI use was marked by the RX service date of the relevant Part D claim and extends the number of days of the medication supply dispensed. The final number of patients with Stage IV RCC undergoing CN who received TKI’s within 90 days of surgery was 82, versus 545 who did not receive TKI treatment. Only patients receiving TKI’s within 30 days of CN were included into the 30-day analyses (n = 37).

### Variables

The assessed outcomes were the 30- and 90-day complication rate, as well as 30- and 90-day overall mortality rate. Complications were identified from inpatient and outpatient claims between the date of nephrectomy and 90 days after surgery using ICD-9 codes from Supplementary Material 1. Covariates included patient’s age at diagnosis (66–69, 70–74, ≥75), gender, race (non-Hispanic white or other), Charlson comorbidity index (0, 1, or ≥2), type of surgery (laparoscopy vs open), histology (clear cell vs non-clear cell) and Fuhrman nuclear grade (1–2, 3–4, or unknown).

### Statistical analysis

Univariate differences between groups were compared using chi-squared tests and standardized differences. To reduce confounding, we matched the TKI cases to controls at a 1:3 ratio using propensity score techniques, resulting in a matched sample of 328 cases total (82 cases and 246 controls). To confirm balance between cases and controls, we again performed both chi-squared tests and calculated standardized differences on the matched sample. The propensity scores were derived from a multivariable logistic model predicting whether a patient received TKIs.

We further compared the adjusted risk for postoperative death and complications between cases and controls using the Cox proportional hazard model. Time 0 was set as the date of nephrectomy and censor time the earliest of the following three: death date, 90 days after nephrectomy, and end of follow-up (December 31, 2010). TKI use was treated as a time-dependent covariate that was turned on only when a patient started using TKI. To achieve a parsimonious model and avoid overfitting, we started the model-fitting with all covariates shown in Table 1, and used a backward selection strategy to eliminate covariates that do not contribute to the model at a stay significance level of 0.1, while keeping the predictor of interest (TKI use) in the model. For all other tests, p values were two-sided and p ≤ 0.05 was considered to indicate statistical significance. All analyses were conducted using SAS 9.4, with the exception of tests on incidence ratios conducted in R using the “epiR” package.

**Table 1:** Demographic, clinical and pathological characteristics of patients that underwent cytoreductive nephrectomy for stage IV RCC by status of perioperative TKI treatment (n = 627).

		All Patients				Matched Controls		
		TKI Users (N = 82)	Controls (N = 545)	Chi-square P Value	Standardized Difference with TKI Users	Controls (N = 246)	Chi-square P Value	Standardized Difference with TKI Users
		Col %	Col %			Col %		
Sex	Male	64.6	62.0	0.65	0.05	60.6	0.60	0.084
	Female	35.4	38.0		—	39.4		—
Age Group	66–69	36.6	29.9	0.25	0.14	39.8	0.76	−0.067
	70–74	32.9	30.3		0.06	33.7		−0.017
	75 or older	30.5	39.8		—	26.4		—
Race	Non-Hispanic White	67.1	80.0	0.008	−0.30	65.9	0.95	0.026
	Other	32.9	20.0		—	34.1		—
Procedure Type	Open	72.0	61.1	0.058	−0.23	72.4	1.00	0.009
	Laparoscopic	28.1	38.9		—	27.6		—
Charlson Comorbidity Index	0	57.3	52.8	0.73	−0.03	61.4	0.74	0.019
	1	24.4	25.9		−0.08	23.6		0.087
	2+	18.3	21.3		—	15.0		—
Grade	1 or 2	25.6	23.1	0.015	0.06	25.2	1.00	0.009
	3 or 4	67.1	56.2		0.23	67.5		−0.009
	Other or Unknown	7.3	20.7		—	7.3		—
Histology	Non-clear cell	42.7	51.0	0.16	0.17	40.7	0.85	−0.04
	Clear cell	57.3	49.0		—	59.3		—

## Results

Table 1 presents demographic and clinicopathologic variables for patients who received TKI’s vs. those who did not receive TKI’s, both prior to and after propensity score matching. Prior to propensity matching, both groups had similar distribution of sex, age, comorbidity level, and clear cell histology. However, patients treated with TKI’s were more likely to be non-white (67.1% vs 80.0%, p = 0.008), undergo laparoscopic nephrectomy (28.1% vs 38.9%, p = 0.058), and have higher grade tumors (67.1% vs 56.2%, p = 0.015). After matching, all Chi-squared tests are statistically insignificant and standardized differences between −0.1 and 0.1.

Ninety-day complication and mortality rates are shown in Table 2. The overall complication rate was 3.25 events per 1000 person-days among patients treated with TKIs and 2.70 among controls. Mortality rate was 1.12 and 1.34 death per 1000 person-days among TKI users and controls, respectively. These measures are not statistically different between the two groups. Differences in specifically defined complications by organ system and mortality rates were also not statistically different.

**Table 2:** 90-day complication and mortality rate (per 1000 person-days)^a^.

Complication	TKI Users (N = 82)		Matched Controls (N = 246)		p-Value	Overall (N = 328)
	Event Count	Event Rate	Event Count	Event Rate		Event Rate
Cardiac	**	0.58	**	0.39	0.53	**
Respiratory	0	0	**	**	>0.1	**
Genitourinary	**	**	**	**	0.75	**
Wound	**	**	**	**	0.39	**
Vascular	**	**	**	**	0.77	**
Miscellaneous Medical	11	1.68	21	1.06	0.21	1.21
Miscellaneous Surgical	0	0	**	**	>0.1	**
Heterologous Blood Transfusion	**	**	23	1.15	>0.1	**
Any Complication Above	20	3.25	50	2.70	0.49	2.84
Death	**	**	28	1.34	>0.1	**

^a^Certain cells of this table are suppressed (**) or coarsened (>0.1) to comply with the Cell Size Suppression Policy of SEER-Medicare and Center for Medicare and Medicaid Services.

Table 3 shows adjusted and unadjusted hazard ratios (HR) from Cox models predicting postoperative complications within 30 and 90 days after surgery. TKI treatment was associated with a higher risk of complications within 30 days (HR = 2.93, 95% CI: 1.17–7.36, p = 0.02), as well as 90 days (HR = 1.84, 95% CI: 1.02–3.32, p = 0.04). Additionally, a Charlson score of 2 or more was also associated with increased complications within both 30 (HR = 2.41, 95% CI: 1.44–4.02, p = 0.001) and 90-days (HR = 2.23, 95% CI: 1.51–3.29, p < 0.0001), compared with those with a Charlson score of 0.

**Table 3:** Hazard Ratios for 30- and 90-day Complication Following Cytoreductive Nephrectomy in patients with Stage IV Renal Cancer.

			30-Day Complication		90-Day Complication	
			HR (95% CI)	p Value	HR (95% CI)	p Value
Unadjusted	TKI (ref = control)		2.73 (1.09, 6.80)	0.03	1.76 (0.98, 3.16)	0.06
Adjusted^a^	TKI (ref = control)		2.93 (1.17, 7.36)	0.02	1.84 (1.02, 3.32)	0.04
	Charlson Comorbidity Index (ref = 0)	1	0.71 (0.36, 1.41)	0.33	0.93 (0.59, 1.47)	0.76
		2	2.41 (1.44, 4.02)	0.001	2.23 (1.51, 3.29)	<0.0001

^a^Covariates eliminated during backward model selection include sex, age group, race, procedure type, grade, and histology.

Table 4 shows adjusted and unadjusted hazard ratios predicting mortality within 30 and 90 days after surgery. The mortality rates among TKI users and controls were not significantly different 30 days nor 90 days from the nephrectomy. However, patients with tumor grade of 3 or 4 (HR = 3.01, 95% CI: 1.37–6.64, p = 0.01) and clear cell histology (HR = 1.71, 95% CI: 1.05–2.79, p = 0.03) had increased risk of 90-day mortality, compared with those with grade 1 or 2 and having non-clear cell histology, respectively.

**Table 4:** Hazard Ratios for 30- and 90-Day Mortality Following Cytoreductive Nephrectomy among Patients with Stage IV Renal Cancer.

			30-Day Mortality		90-Day Mortality	
			HR (95% CI)	p value	HR (95% CI)	p value
Unadjusted	TKI (ref = control)		1.37 (0.18, 10.22)	0.76	1.42 (0.67, 2.99)	0.36
Adjusted^a,b^	TKI (ref = control)		1.37 (0.18, 10.22)	0.76	1.47 (0.70, 3.12)	0.31
	Histology (ref = non-clear cell)	Clear cell	—	—	1.71 (1.05, 2.79)	0.03
	Grade (ref = 1, 2)	3–4	—	—	3.01 (1.37, 6.64)	0.01
		Unknown	—	—	2.24 (0.89, 5.62)	0.09

^a^Covariates eliminated during backward model selection include sex, age group, race, procedure type, and Charlson comorbidity index.

^b^In model for 30-day mortality, histology and grade were also eliminated from the model. No covariate was significant at a stay significance level of 0.1. Therefore, the adjusted model is identical to the unadjusted model.

## Discussion

Targeted therapies have changed the treatment paradigms of metastatic RCC significantly over the last decade. Two recent population-based studies showed that the treatment of metastatic RCC with TKI’s is disseminated across the US and might increase overall survival^5,14^, therefore leading to a growing interest in combining CN with TKI treatment.

Our results show that perioperative TKI treatment is independently associated with increased risk of 30- and 90-day overall complication rates following CN. Moreover, we demonstrated that higher Charlson comorbidity index is another independent risk factor for postoperative complications (Table 3). TKIs can cause gastrointestinal, cardiovascular and hematologic toxicity^15^. Fifty percent of patients treated with sorafenib or sunitinib experience diarrhea or nausea, but these side effects are usually mild and do not require treatment discontinuation^15^. Sunitinib and sorafenib can also induce hypertension, decrease left ventricular ejection fraction and prolong QTc-interval. Recently, Jang *et al*.^16^ showed that patients older than 65 years old with metastatic RCC treated with TKIs had an increased risk of cardiovascular events, particularly stroke. Previous studies showed that there was a higher incidence of intracerebral hemorrhage (ICH) and deaths associated with ICH in patients with RCC brain metastasis treated with TKIs and delayed wound healing associated with VEGF inhibition^17^. However, other retrospective studies comparing patients who were treated with TKIs before and after CN did not show an increase in complications with TKI treatment. Margulis and collaborators evaluated surgical parameters and perioperative complications in patients treated with TKIs prior to CN and compared them with patients treated with up-front CN^18^. Their results did not show significant differences in surgical parameters, incidence of perioperative complications and mortality in patients treated with TKIs. Another multi-institutional retrospective analysis of patients with mRCC compared patients who underwent CN followed by adjuvant sunitinib with patients that were initially treated with sunitinib followed by either CN if had partial response or stable disease^19^. The overall complication rates were not statistically significant different between the two groups, but the patients treated with TKIs followed by CN had significantly higher rates of Clavien-Dindo grade III/IV complications. Our results did not show a significant increase in blood transfusion rates or wound- and vascular-related complications after CN in patients treated with TKIs (Table 2) consistent with results from prior studies with smaller number of patients^12^. Overall TKIs are associated with adverse effects that in the postoperative period might exacerbate underlying medical conditions in sicker patients leading to worst outcomes in this specific patient population. In fact, this may explain the lack of survival benefit in the CARMENA trial that enrolled mainly poor-risk patients.

CARMENA and SURTIME are the two randomized clinical trial in this topic that evaluated the survival benefit and surgery-related morbidity of CN in combination with TKIs. However, SURTIME accrual was poor with loss of enrolment sites over time, the study was downsized and the end points revised. CARMENA was also underpowered, patients were not categorized according to the IMDC-risk categories^20^, and approximately 40% of patients’ tumor burden measured by RECIST v1.1 was metastatic, making this RCC patient population a suboptimal one to answer the question about potential benefits of CN^8^.

Our results also show that perioperative TKI use does not significantly affect overall mortality in patients with metastatic RCC. Interestingly, higher Fuhrman nuclear grade and clear cell histology were not significantly associated with higher postoperative complications but were independent risk factors for 90 days overall mortality. Therefore, tumor characteristics and aggressive tumor biology are not associated with surgical complications but drives metastatic RCC prognosis independently of TKI treatment. These findings are important for planning a careful multidisciplinary approach to metastatic RCC.

Several study limitations affect the interpretation of our results. This is a retrospective analysis of a population-based database composed of older patients covered by Medicare, that does not have enough granularity to assess the dose and frequency of TKI administration, and primary tumor characteristics besides grade and histology. Thus, results from SEER-Medicare population might not be applicable to the entire US population. Furthermore, only 82 patients were perioperatively treated with TKIs, which impairs a more comprehensive analysis and determination of increased risk for specific complications. However, SEER-Medicare captures a heterogeneous health care system and patient population with less stringent treatment criteria than patients enrolled in clinical trials.

In conclusion, as both SURTIME and CARMENA studies were unable to clarify the role of CN in healthy patients with limited metastatic burden, CN remains a valuable treatment option in this patient population. If utilizing TKI therapy in these patients in conjunction with CN, our results should inform surgeons of the possibility of increase CN complication rates in this setting, especially in comorbid patients in which the role of CN is in question. Further studies are needed to clarify optimal treatment protocols, including adjuvant vs. neoadjuvant therapies in conjunction with CN, for patients with metastatic RCC.

## Supplementary information

Supplementary information
