# Supplementary material for: Complications associated with perioperative use of tyrosine kinase inhibitor in cytoreductive nephrectomy
Source: Sci Rep. 2019 Oct 24;9:15272. doi: 10.1038/s41598-019-51548-4 (PMC6813342; doi:10.1038/s41598-019-51548-4)
Supplement: Supplementary file 1 — Supplementary information [file 41598_2019_51548_MOESM1_ESM.docx]

Supplementary material 1. ICD-9 Diagnosis, Procedure and CPT Codes used to identify complications after cytoreductive nephrectomy (Adopted from Hu et al. [1]).

| **Category** | **Diagnosis Codes** | **Procedure Codes** |
| --- | --- | --- |
| Cardiac | ICD9: 410.xx, 402.01, 402.11, 402.91, 428.xx, 427.5, 997.1 |  |
| Respiratory | ICD9: 518.0, 514, 518.4, 466.xx, 480.xx, 481, 482.xx, 483.xx, 485, 486, 518.5, 518.81, 518.82, 799.1, 997.3 |  |
| Genitourinary | ICD9: 590.1x, 590.2, 590.8x, 590.9, 591, 593.3, 593.4, 593.5, 593.81, 593.82, 595.89, 596.1, 596.2, 596.6, 997.5 | ICD9: 55.02, 55.03, 55.12, 55.93, 55.94, 59.93, 97.61, 97.62, 56.1, 56.41, 56.74, 56.75, 56.81, 56.84, 56.86, 56.89, 56.91; |
|  |  | CPT: 50040, 50120, 50125, 50395, 50398, 50605, 52290, 52332, 52334, 50600, 50700, 50715, 50760, 50770, 50780, 50782, 50783, 50785, 50800, 50810, 50815, 50820, 50825, 50840, 50900, 50940 |
| Wound | ICD9: 567.xx, 998.3, 998.5x, 998.6 | ICD9: 54.61, 54.1x, 54.91, 54.0, 59.19 |
|  |  | CPT: 26990, 45020, 49060, 51080 |
| Vascular | ICD9: 415.1, 451.1x, 451.2, 451.81, 451.9, 453.8, 453.9, 997.2, 999.2, 444.22, 444.81,  433.xx, 434.xx, 436, 437.xx |  |
| Miscellaneous Medical | ICD9: 584.xx, 586, 785.5x, 995.0, 995.4, 998.0, 999.4, 999.5, 999.6, 999.7, 999.8, 457.8, 560.1, 560.8x, 560.9, 997.4, 353.0, 354.2, 723.4, 955.1, 955.3, 955.7, 955.8, 955.9, 593.4, 531.xx, 532.xx, 533.xx, 782.4, 573.8 |  |
| Miscellaneous Surgical | ICD9: 599.1, 596.1, 596.6, 565.1, 569.3, 569.83, 569.4x, 998.1x, 998.83, 998.9, 998.2, 998.4, 998.7, 604.0, E870.0, E870.4,  E870.7, E870.8, E870.9, E871.0, E873.0, E876.0, 956.0, 956.1, 956.4, 956.5, 956.8, 956.9, 902.50, 902.51, 902.52, 902.53, 902.54, 902.59 | ICD9: 46.03, 46.04, 46.10, 46.11, 46.14, 48.4x, 48.5, 48.6x, 48.7x, 48.9x |
| Heterologous Blood  Transfusion |  | ICD9: 99.04;  CPT: 86930, 86965, 86999;  HCPCS: P9010, P9011, P9017, P9021, P9022, P9038, P9039, P9040 |

Reference

1. Hu JC, Gu X, Lipsitz SR, Barry MJ, D'Amico AV, Weinberg AC, Keating NL. Comparative effectiveness of minimally invasive vs open radical prostatectomy. *JAMA*. 2009; 302: 1557-64.
